# Novel drug delivery systems of Chinese medicine for the treatment of inflammatory bowel disease

**DOI:** 10.1186/s13020-019-0245-x

**Published:** 2019-06-17

**Authors:** Caifang Gao, Lijuan Liu, Yangyang Zhou, Zhaoxiang Bian, Shengpeng Wang, Yitao Wang

**Affiliations:** 1 State Key Laboratory of Quality Research in Chinese Medicine, Institute of Chinese Medical Sciences, University of Macau, Avenida da Universidade, Taipa, Macao, SAR China; 2 PU-UM Innovative Institute of Chinese Medical Sciences, Guangdong-Macau Traditional Chinese Medicine Technology Industrial Park Development Co., Ltd, Hengqin New Area, Zhuhai, China; 3 College of Pharmacy, Chengdu University of Traditional Chinese Medicine, Chengdu, Sichuan China; 4 School of Chinese Medicine, Hong Kong Baptist University, Kowloon, Hong Kong, SAR China; 5 Leiden University European Center for Chinese Medicine and Natural Compounds, Institute of Biology, Leiden University, Leiden, The Netherlands

**Keywords:** Inflammatory bowel disease, Chinese medicine, Drug delivery system, Ulcerative colitis

## Abstract

Inflammatory bowel disease (IBD) is an idiopathic intestinal inflammatory disease that comprises ulcerative colitis (UC) and Crohn’s disease (CD). IBD involves the ileum, rectum, and colon, and common clinical manifestations of IBD are diarrhea, abdominal pain, and even bloody stools. Currently, non-steroidal anti-inflammatory drugs, glucocorticoids, and immunosuppressive agents are used for the treatment of IBD, while their clinical application is severely limited due to unwanted side effects. Chinese medicine (CM) is appealing more and more attention and investigation for the treatment of IBD owing to the potent anti-inflammation pharmacological efficacy and high acceptance by patients. In recent years, novel drug delivery systems are introduced apace to encapsulate CM and many CM-derived active constituents in order to improve solubility, stability and targeting ability. In this review, advanced drug delivery systems developed in the past and present to deliver CM for the treatment of IBD are summarized and future directions are discussed.

## Background

Inflammatory bowel disease (IBD) is a chronic and multifactorial inflammatory disorder of the intestinal tract, associating with an immunological imbalance of the intestinal mucosa [1]. IBD is commonly known to be classified into two major subtypes, namely ulcerative colitis (UC) and Crohn’s disease (CD). UC is a continuous inflammation of the colonic mucosa, while CD is a non-continuous whole-layer inflammation and can affect any region of the whole gastrointestinal tract (GIT) from the mouth to the anus [2, 3]. Typically, clinical manifestations of IBD are characterized as abdominal pain, diarrhea, weight loss, and even bloody stools. Moreover, a relentless climb in incidence has been observed among the younger in recent decades [4]. With the development and progression of IBD, various complications and other conditions occasionally develop, such as stenosis, fistula and colitis-associated cancer. Given its early onset, chronic nature and the need for treatment till the end of life, the cost of medical treatment for IBD would be enormous and impose a significant economic burden on IBD patients.

Historically, patients with IBD were treated with non-biological therapies, containing 5-aminosalicylic acid (5-ASA), antibiotics, and in some cases, steroids. Among these conventional therapies, corticosteroids are considered to be the most effective therapies, providing symptomatic improvement for patients with IBD [5]. However, with the emerging side effect and steroid-dependent remission by non-biological treatment, new biological therapies have been clinically developed, such as anti-tumor necrosis factor (TNF) agents (infliximab, adalimumab, and certolizumab), anti-adhesion molecules (vedolizumab), some blockage of downstream signaling (tofacitinib), proinflammatory cytokines (ustekinumab), and others agents like ozanimod [6–10]. Although these new biological therapies have been reported to be well tolerated, their effectiveness in treating all patients is moderate and the safety problems associated with risks of GIT ulcers, bleeding and perforation, metabolic disorders or myelosuppression have become more and more conspicuous [11, 12]. Therefore, the development of effective and low-cost therapeutics is of high importance and necessity for patients with IBD.

Due to limited effective medications and concerns of side effects, the estimated prevalence of patients with IBD in North America and Europe using complementary and alternative medicines (CAM) has ranged from 21 to 60%, ranking among the highest users of CAM [13, 14]. Chinese medicine (CM) as one of the most developed branches of CAM has been widely used to treat IBD. Substantial studies have revealed that CM and many CM-derived active constituents, such as rutin, quercetin, resveratrol, curcumin and berberine, can effectively reduce the intestinal inflammation and promote the wound healing through multiple mechanisms [15, 16]. Although the application of a majority of CM or their extracts has achieved decent therapeutic efficacy in experimental IBD models [17–20], the widely use of CM is severely limited because of poor water-solubility, instability at different environmental factors, and short half-life.

Novel drug delivery systems (DDS), which enclose therapeutic drugs in different preparations through a series of nanotechnology and excipients, have revolutionized the treatment of IBD in recent years. Novel DDS can be engineered to improve the solubility and stability, increase the bioavailability, control the drug release rate, increase the accumulated concentration at the desired site and reduce the systemic toxicity of the drugs [21]. Varieties of DDS are designed according to the physiological and pathological features of IBD, which is characterized by the change of pH, gastric empty time, unique enzymes and micro-flora, higher pressure and overexpression of specific proteins. The emergence of novel DDS has provided new platforms for CM to exert their potentials in treatment of IBD and relieve the related symptoms of IBD [22, 23]. Here, the rationale of this review is to summarize the DDS developed in the past and present to deliver CM for the treatment of IBD.

## The complex pathogenic mechanisms of IBD

Nowadays, IBD is recognized as a chronic relapsing intestinal inflammation with unknown etiology and pathogenesis. People with IBD could suffer from the course of episodic or persistent symptoms, as well as recurring bowel trouble. Generally, it is worldwide accepted that a complex interaction between the genetic, environmental, microbial and immunological factors involves in IBD pathogenesis [24]. Among four components, immune disorders are considered to be the most essential factor contributing to the development of IBD. The dysregulation of intestinal epithelial barrier indicates the incidence of IBD.

Over the past decades, rapid successes in genetic analysis and sequencing uncover more and more IBD-related/causing genes and pathways. These genes and signaling pathways mainly implicated in the maintenance of intestinal homeostasis, such as mucus barrier (*GNA12*, *HNF4A*, *CDH1*, *MUC19*), epithelial restitution (*REL*, *PTGER4*, *STAT3*, *ERRFI1*), regulation of innate immune (*CCL11*, *CCL2*, *CCL7*, *MST1*), autophagy (*ATG16L1*, *XBP1*, *NOD2*, *LRRK2*, *CUL2, IRGM*) and adaptive immunity (*NDFIP1*, *TNFSF8*, *TAGAP*, *IL2*, *IFNG*, *IL5*, *IL7R*, *IRF5*, *IL10*, *IL27*, *CREM*), IL-23/T_H_17 signaling (*IL23R, JAK2*, *IL21*, *TNFSF15*), endoplasmic reticulum (ER) stress (*CPEB4*, *ORMDL3*, *SERINC3*, *XBP1*) [25]. Recently, the largest genetic association studies and transethnic analysis documented about 200 susceptibility loci and over 300 potential genes for IBD [26]. However, these identified genetic factors and susceptibility loci disclosed so far account for only 20–25% of the genetic risk of IBD. In addition, several environmental factors play risky roles in the pathogenesis of IBD, including smoking, diet, some nonsteroidal anti-inflammatory drugs (NSAIDs), antibiotics, social stress or psychological element [24]. Although the relationships among them remain poorly understood, accumulating evidences have shown that smoking and drugs, including NSAIDs and antibiotic, have significant impacts on triggering onset or relapse of IBD, while high levels of stress and the psychological changes from perceived stress could partly medicate the deterioration of IBD [27–32]. Furthermore, many studies have established the association between the changes in intestinal microbiota and IBD. A significant reduction in biodiversity and stability of gut microbiota and a marked increase in adherent and invasive bacteria have been found in patients with IBD in comparison with that of healthy people [33–36].

Currently, more and more efforts have been dominated in investigating the relationships between gut inflammation and IBD pathogenesis. The dysfunctions of innate and adaptive immune signaling have been well-documented to play crucial roles in contributing to the abnormalities of gut inflammatory response in IBD. Immunological studies focused on innate immune responses in IBD show the importance of epithelial barrier integrity, innate microbial sensing and autophagy in contributing to IBD pathogenesis [37–40]. On the other hand, most recent studies indicated that CD has been associated with an aberrant Th1 response-driven gut inflammation while UC has been mediated by a non-conventional Th2 immune response in gut when it comes to an adaptive immune response in IBD [41]. Taken together, these components are responsible for IBD, which facilitated by defects in the intestinal epithelial barrier, causing innate and adaptive immune response with a large amount of cytokines, and then leading to the activation of subclinical or acute mucosal immune system, eventually resulting in an active and chronic inflammation and tissue destruction like fibrosis, abscess, fistula, and even cancers.

## Therapeutic potential of CM for the treatment of IBD

In recent years, various CM and CM-derived active constituents have been employed in the treatment of IBD and displayed great potential and effectiveness from bench to clinical application. It has been reported that CM can be used to treat a series of acute and chronic gastrointestinal diseases by clearing body heat and dampness, detoxifying, and invigorating the circulation of blood [42, 43]. Notably, CM is appealing more and more attention and investigation in the treatment of IBD due to the potential benefits of high acceptance by patients, less undesirable side effects, and relatively low cost. The existing CM for the treatment of IBD can be classified according to preclinical drugs, drugs in clinical trials, and listed drugs. Some of the drugs currently in preclinical studies are described below, and there are many others that have not been mentioned in this article. Periplaneta Americana, a medicinal insect, its ethanol extract could attenuate the dextran sulfate sodium (DSS)-induced UC in rats, by means of ameliorating intestinal inflammation, improving intestinal barrier function, and regulating the disturbed gut microbiota, modulating the flora structure, and restoring the intestinal immune system [44]. *Wedelia chinensis*, the whole grass of *Wedelia chinensis* (Osbeck) Merr, can significantly ameliorate the symptoms of colitis in animal models, such as diarrhea, rectal bleeding and weight loss, reduce colonic atrophy and histopathological damage caused by inflammation [45]. Tanshinone IIA, the major active lipophilic components of Danshen, and Notoginsenoside R1, the main bioactive component of Panax Notoginseng, could both attenuate experimental inflammatory bowel disease via pregnane X receptor activation [46, 47]. Several CM products have been introduced to trials of IBD patients after passing animal studies successfully. In UC, aloe vera gel, tormentil extracts, wheat grass juice (*Triticum aestivum*), *Andrographis paniculata* extract (HMPL-004) and topical Xilei-san were better to placebo in inducing remission or response, and curcumin was superior to placebo in maintaining remission [48–53]. In CD, *Artemisia absinthium* (wormwood) and *Tripterygium wilfordii* were superior to placebo in inducing remission and preventing clinical recurrence of post-operative CD respectively [54, 55]. A multicenter study showed that the efficacy of *Andrographis paniculata* (HMPL-004) was as potent as mesalazine after an 8-week therapy in mild-to-moderate UC patients, thus indicating that HMPL-004 is a promising alternative to mesalazine in UC [56]. In a randomized, placebo-controlled trial, 39 adult postoperative CD patients were treated with *Tripterygium wilfordii* (TW) or SASP for 2 weeks. The recurrence rate of CD in the TW treated group was significantly lower than that in the SASP-treated group [54]. CM that have been introduced into the market for the treatment of IBD are mainly compound preparations, including Gubenyichang Tablets, Bupiyichang Pills, Guchang Zhixie Pills, Changweining Tablets and Shenqi Baizhu Pills, etc. Therefore, the therapeutic potential of CM in the IBD therapy is large and the development of advanced CM or CM-derived formulations is promising.

## Novel DDS of CM for the treatment of IBD

Substantial CM are found to have potent anti-inflammatory effects, but their wide application into clinic was challenged due to poor water-soluble ability, instability and rapid metabolism. Fortunately, nanotechnology and related technologies advanced the development of novel DDS and thus addressed these problems to some extent. In this section, the following DDS were introduced to overcome undesirable features of CM, such as nanoparticle, self-nanoemulsifying DDS (SNEDDS), nanoemulsion, nanosphere, nanotube, solid lipid microparticle, capsule and lipid-based nanocarriers.

### pH-dependent DDS

The pH of GIT is elevated from the stomach to the colon. The pH value of the colon can be achieved 7.0–7.4 and this character can be exploited to design a pH-dependent DDS to specifically release the drug in the colon inflammation site [57]. The most commonly used carrier material for pH-dependent drug carriers is the acrylate copolymer (Eudragit). It is an anionic polymer in which the carboxylic acid group does not dissociate at a low pH and is therefore insoluble in the stomach. After entering the small intestine, the polymer molecules become ionized and gradually dissolve as the pH increases [58]. The greater the proportion of carboxyl groups in the molecule, the higher the pH required for dissolution. Several Eudragit-based products have been approved as pharmaceutical excipients to achieve pH-dependent drug release in the colon, currently.

Silybin is a flavonolignan and extracted from the seeds of *Silybum marianum* L. Since ancient times, it has been used for the treatment of various gastrointestinal and liver diseases, which is attributed to its radical scavenging activities [59]. Eudragit RL PO NPs were prepared using solvent evaporation emulsification technique and then were coated by Eudragit FS30D to deliver silybin in the inflamed intestinal site. Animal experimental results demonstrated the significant reduction of TNF-α, IL-6 and myeloperoxidase (MPO) activity and the amelioration of macroscopic and histopathological scores by the optimized NPs in the acetic acid-induced rat colitis model compared to control group [60].

Curcumin is a bioactive polyphenol isolated from the rhizome of the *Curcuma longa* L. (turmeric) [61, 62]. Extensive researches on curcumin have proven that it is a molecule with anti-oxidant, anti-inflammatory and antitumorigenic properties [63–65]. As a BCS IV drugs, the solubility and permeability of curcumin are both poor. The microsponge of curcumin was developed by quasi emulsion solvent diffusion method. Release studies revealed that microsponges prevented the premature release of curcumin in upper GIT and specifically released curcumin at colonic pH. Pharmacodynamic study demonstrated that, compared to free curcumin, curcumin microsponges can ameliorate edema, necrosis, and hemorrhage of colon. In addition to microsponge, curcumin-loaded poly (lactic-*co*-glycolic acid) (PLGA) NP/microparticle and microsphere (zein and PVMMA) [66–68] were prepared and then coated with pH-sensitive materials Eudragit S100 and FS30D. In vitro and in vivo assays of the three curcumin-loaded DDS exhibited pH-dependent release behavior and delivered curcumin to the colon lesion to exert excellent anti-inflammation efficacy [69].

Rutin is a citrus flavonoid glycoside found in many plants. Like other flavonoid compounds, rutin possesses strong anti-oxidant activity. So starch and acrylic acid were copolymerized using the direct gamma radiation technique to form the hydrogel and then encapsulated rutin. In vitro release of rutin-loaded hydrogel revealed strong pH-dependent release behavior. The reduction of colon/body weight ratio, MPO activity, TNF-α, nitric oxide and histopathological results confirmed the efficacy of the poly (starch/acrylic acid) hydrogel loaded rutin [70]. Ellagic acid, a natural phenol, has the antiproliferative and antioxidant properties. The microsphere of ellagic acid was prepared using a pH-sensitive polymer, Eudragit P-4135F, carmellose sodium and Span 80. Release behavior suggested that the pH-responsive microsphere was prepared successfully and can be used to deliver drug in the colon for the treatment of IBD [71].

Shikimic acid (SA), more commonly known as its anionic form shikimate, affects arachidonic acid metabolism, inhibits platelet aggregation, inhibits arterial and venous thrombosis, and has anti-inflammatory effects. Butyric acid (BA), a short chain fatty acid, has good therapeutic effects on UC confirmed by many experiments. But the short half-life of oral administration limited its application. 3,4,5-Tributyryl shikimic acid (TBS), a novel prodrug, was synthesized through an ester bond between SA and BA. Amberlite 717, the anion-exchange resin, was employed as the carrier to encapsulate TBS through the ion-exchange reaction. Simultaneously, Eudragit S100, the enteric coating material, was introduced to encapsulate the drug-loaded resin to form the drug-loaded resin microcapsule (TBSS-DRM). The TBSS-DRM exhibited a good therapeutic effect on 2,4,6-trinitrobenzenesulfonic acid (TNBS)-induced experimental colitis mouse [72]. Rectal administration of Piceatannol, an analog and metabolite of resveratrol, by colon-targeted capsule also ameliorated rat colitis and reproduced the molecular effects in the inflamed colonic tissues [73].

### Time-delayed and pressure-dependent DDS

Although the gastric emptying time is extremely irregular, the transit time of the material in the small intestine is relatively constant, usually 3 to 4 h [74]. Based on this feature, a time-delayed colon targeting system was designed to ensure that the drug began to release after 3 to 4 h of leaving the stomach. At present, it is common to encapsulate drugs with poorly soluble coating materials that are difficult to decompose. Adjusting the proportion and amount of material is used to control drug release time.

Acetylharpagide, extracted from *Ajuga decumbens*, is widely used for remedying infectious and inflammatory diseases in Southern China. But it can be destroyed by the stomach acid due to the hemiaceta structure. Acetylharpagide tablets were formulated with the dual mechanism, pH-dependency and time-delay release. The core tablets were coated with the ethyl cellulose and suitable channeling agent followed by coating with pH dependent polymers. In vitro drug release and the pharmacokinetic evaluation in dogs showed that acetylharpagide colon-targeted tablets could target the drug to the colon [75].

There are peristaltic waves in the GIT of the body, but a large amount of small intestinal fluid in the small intestine can effectively buffer the intracavity pressure, and the intracavitary pressure of the intestinal contents is reduced. However, the colon absorbs a large amount of liquid, the contents solidify, and the intracavitary pressure is increased in the colon [76]. Pressure-controlled DDS are constructed to bear up against luminal pressure in the small intestine, but to collapse at the higher colonic pressure with a final drug release in the colon 3–7 h after peroral administration. Usually, the preparation of pressure-dependent DDS is based on the coating of gelatine capsules with ethyl cellulose. To date, there is no report about the pressure-dependent DDS for the delivery of CM to treat IBD.

Variation in GI transit times, altered gastro-intestinal motility and water absorption in IBD may limit the therapeutic benefits of these two types DDS. The change of transit times and pressure in IBD patients should be furtherly investigated and discussed to guide a definite direction. More pharmaceutical investigations for time-delayed and pressure-dependent DDS of CM are essential to evaluate the therapeutic potential for the therapy of IBD.

### Enzyme/microbiota-activated DDS

The human colon begins with the cecum and ends with the rectum. The physiological structure is divided into four parts, namely ascending colon, transverse colon, descending colon and sigmoid colon. The colon is mainly used to absorb water and electrolytes, store the body’s metabolites, solidify them into feces, and excrete them through the rectum. At the same time, the colon also provides a suitable living environment for the inside microorganisms. Studies have shown that the colon is rich in more than 400 beneficial bacteria, and the bacteria in the colon fluid is about 1 × 10^11^/mL, which forms a very large flora gradient with the small intestine. In the colon, these bacteria can produce a large number of highly active proteolytic enzymes and peptidases, which can catalyze many metabolic reactions. The anaerobic environment in which the colon is located makes the enzymatic reaction at the colon mainly degradation [77–79]. Using a material that can be degraded by enzymes specific to the colon (such as azo degrading enzyme, glycosidase, pectinase, etc.) as a targeting carrier material, the formulation is degraded and the drug can be released and then absorbed by the colon site to increase the bioavailability of the drugs.

Rutin pellets were successfully prepared and showed very good characteristics. Pellets presented desirable rutin dissolution profiles and excellent stability when coated with sodium alginate/chitosan. The combination of rutin chitosan, sodium alginate and pellets could form a promising preparation free of side effects for life-long therapy of IBD [80].

Resveratrol is also a polyphenol bioactive agent present in vegetables, fruits and plants and it has various biological activities, such as antitumor, antiviral and antioxidant effect [81]. However, rapid absorption and extensive metabolism in the liver and gastrointestinal tract lead to its low oral bioavailability [82]. Ca-pectinate beads were designed to deliver resveratrol in the treatment of IBD. Animal experiments showed that the lowest disease activity index (DAI) and histopathological score have been recorded in the group treated by Ca-pectinate beads and the anti-inflammatory effects of resveratrol could be attributed to its inhibitory effect on sphingosinekinase 1 (SphK1) [83].

Icariin, isolated from CM Horny Goat Weed (*Epimedium alpinum* L.), is commonly used by mouth for sexual performance problems and inflammation-related diseases. The poor solubility and low bioavailability limited its application in the clinic [84]. Alginate–chitosan microspheres loaded with icariin were investigated in TNBS/ethanol-induced colonic mucosal injury rats. Pharmacodynamics studies indicated that it could not only reduce the colonic injury, but also inhibit the inflammatory response in colonic mucosa [85].

To achieve colon targeted delivery, chitosan and nutriose were coated on the surface of the quercetin nanovesicles. A marked amelioration of symptoms of TNBS-induced colitis was observed in animals treated with quercetin-loaded coated vesicles, favoring the restoration of physiological conditions [86].

**Fig. 1:**
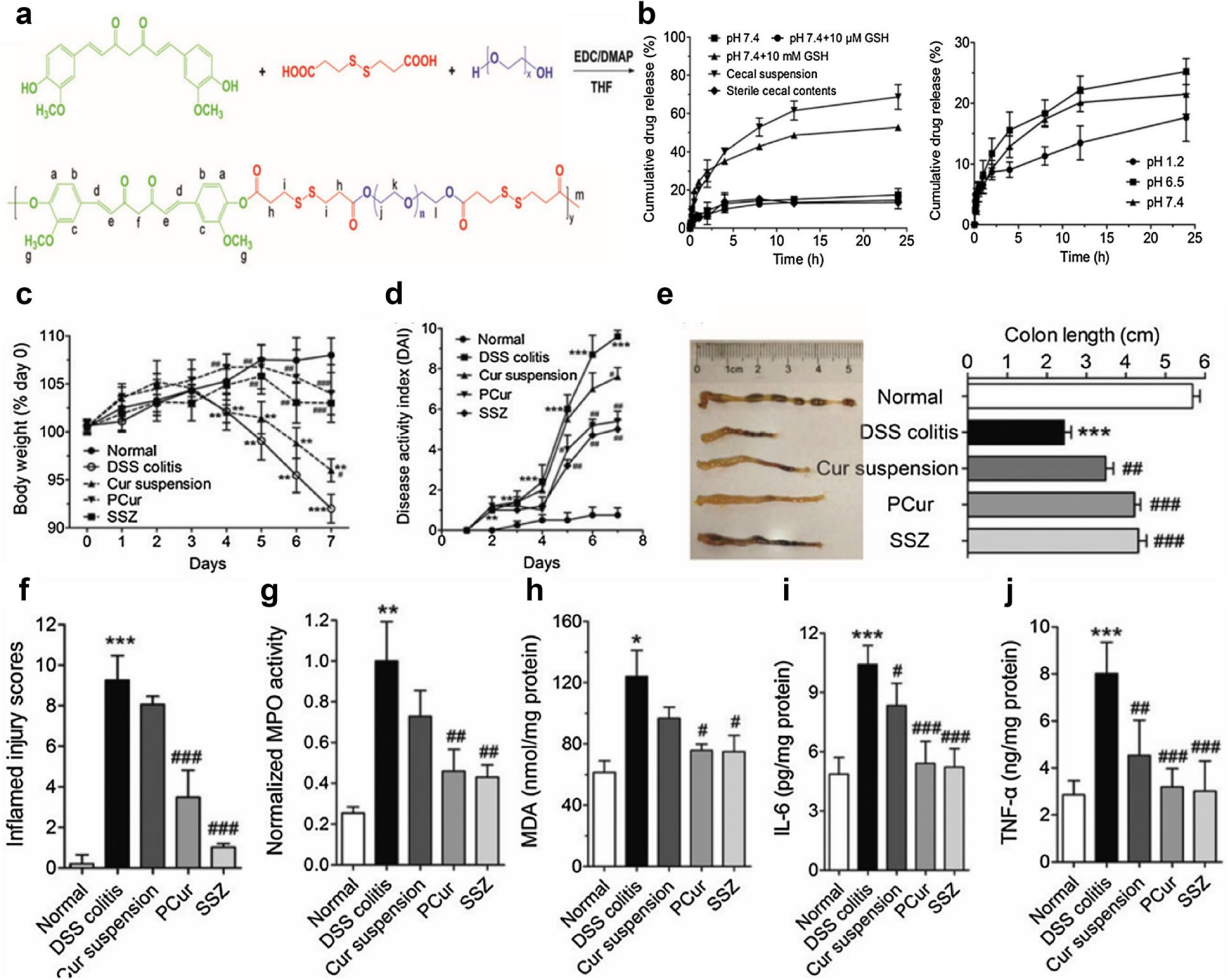
Polycurcumin conjugate for the treatment of IBD. **a** Synthetic scheme of polycurcumin conjugate. **b** In vitro release behavior in the different conditions. **c** Change in body weight, **d** DAI evaluation. **e** Colon length of normal mice and DSS-induced mice receiving different treatments. **f** Quantitative scores of inflamed degree. **g** MPO activity, **h** MDA. **i** IL-6 and **j** TNF-α levels in colonic tissues after administration of polycurcumin (Reprinted with permission from Ref. [87]. Copyright Taylor and Francis Online 2016)

Curcumin polymer (polycurcumin, PCur) was synthesized through disulfide bond between hydrophilic PEG and hydrophobic curcumin (Fig. 1). PCur led to preferential accumulation of curcumin in the inflamed regions owing to the good solubility, proper size and neutral properties. Moreover, from the release curve, we can observe a significantly elevated release of curcumin when responding to a bacterial reduction in the colon. Finally, in DSS-induced murine model of IBD, orally administered PCur ameliorated the inflammatory progression and could protect mice from IBD [87]. Novel polyacrylamide-grafted-xanthan gum (PAAm-g-XG) NPs were prepared for colonic delivery of curcumin. Release studies indicated microflora-dependent drug release property of NPs. Curcumin NPs reduced nitrite and myeloperoxidase levels, prevented weight loss and attenuated colonic inflammation in acetic acid-induced IBD in rats [88].

Sinomenine, a pure alkaloid extracted from *Sinomenium acutum* (Thumb.) Rehd. et Wils., is widely known for its anti-inflammatory and immunosuppressive effects [89]. Due to its low oral bioavailability, chitosan microspheres coated by Eudragit were prepared to delivery sinomenine to the colon lesion for IBD therapy. In DSS-induced experimental colitis, DAI of the sinomenine enteric-coated microspheres-treated group was significantly lower than that of the SASP-treated group, which might be attributed to the suppression of the TLR/NF-κB signaling [90]. Similarly, curcumin (CN)-containing chitosan NPs (CS-NPs) coated with Eudragit FS30D were prepared by ionic gelation and solvent evaporation method. In vivo distribution revealed good accumulation of CS-NPs in the colonic region [91].

From the above examples, it is not difficult to see that chitosan and sodium alginate are used as the main carrier materials of enzyme-sensitive (mucoadhesive) colon-targeted DDS to deliver CM for the treatment of IBD. Therefore, for the specific azo reductase and disulfide reductase in the colon, the DDS by connecting disulfide bonds and azo bonds between the drug and the carrier would be a good choice to treat IBD.

### Co-delivery systems for the treatment of IBD

**Fig. 2:**
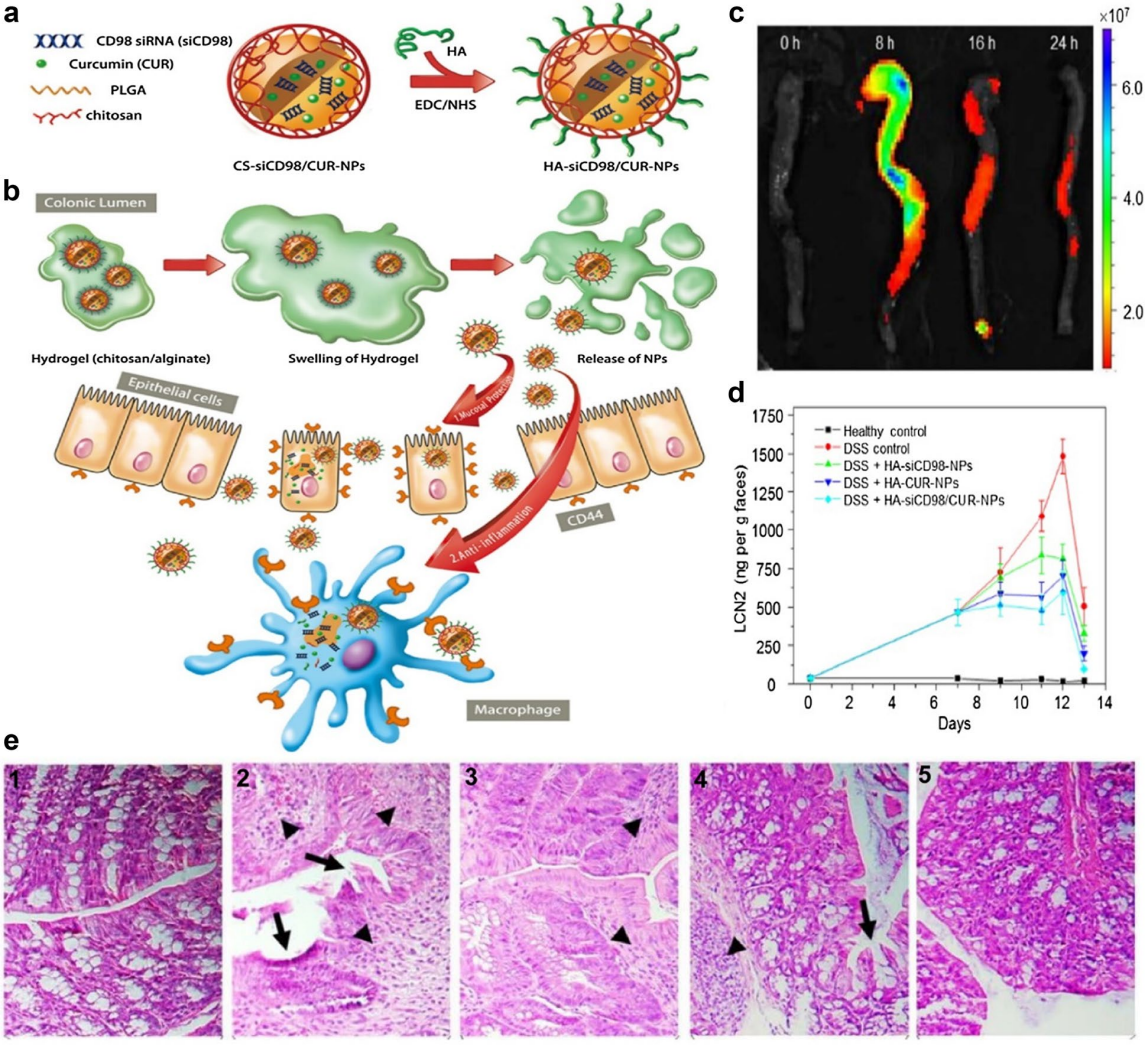
Orally targeted HA-siCD98/CUR-NPs against UC through synergistic effects. **a** The fabrication process of HA-siCD98/CUR-NPs. **b** Scheme illustration of HA-siCD98/CUR-NPs embedded in hydrogel (chitosan/alginate) in vivo. **c** Accumulation of HA-functionalized NPs embedded in hydrogel in colon at four different time points after oral administration. **d** The concentration of fecal Lcn-2 and **e** H&E-stained colon sections of mice treated with different NPs for 6 days, Healthy control (1), DSS control (2), DSS + HA-siCD98-NPs (3), DSS + HA-CUR-NPs (4) and DSS + HA-siCD98/CUR-NPs (5) (Reprinted with permission from Ref. [92]. Copyright Ivyspring International Publisher 2016)

Co-delivery of different kinds of drugs can overcome the unsatisfactory disadvantage and improve therapeutic efficacy through synergistic interaction. CD98 is highly overexpressed on epithelial cells and macrophages in the colon tissue under mucosal damage and inflammation. Previous research has proved that siRNA targeting CD98 (siCD98) could decrease the severity of UC by down-regulating the expression of CD98 in colitis tissue. Hyaluronic acid (HA)-functionalized NPs can realize targeted delivery of siCD98 and curcumin to colonic epithelial cells and macrophages (Fig. 2). Furthermore, siCD98 and curcumin can synergistically prevent mucosal damage and reduce inflammation [92].

Piperine (PIP), an important bioactive compound of black pepper, could inhibit metabolizing enzymes, retard clearance rate of *P*-glycoprotein (P-gp) efflux pump and downregulate the expression of NF-κB, thus enhancing the absorption of curcumin and protecting against oxidative damage. Treatment with CUR-PIP-SMEDDS has an equivalent effect to 5-ASA in maintaining remission of UC by retention enema administration. In addition, it could directly interact with the inflamed epithelium tissue of the mice colon and release curcumin immediately to increase the local concentration in colonic lesion site [93]. In a similar way, thiolated chitosan/alginate composite microparticulates (CMPs) coated by Eudragit S-100 were developed for colon-specific delivery of 5-ASA and curcumin. The combination of pH-sensitive, mucoadhesion and controlled delivery properties could specifically deliver 5-ASA and curcumin to the site of the colon, and markedly alleviate the colon inflammation of colitis rats [94].

Celecoxib, belonging to the nonsteroidal anti-inflammatory drug, is widely used for the treatment of inflammation through inhibiting cyclooxygenase-2 (COX-2) enzyme. But the bioavailability of the drug is poor and it has severe cardiac, gastric and renal toxicity. pH-sensitive NPs loading curcumin and celecoxib were prepared by solvent emulsification evaporation method. This local delivery would be more effectively taken up by the colonic milieu, improve their solubility and bioavailability and circumvent the other organ toxicity of celecoxib [95].

### Other colon-targeted DDS

Apart from above mentioned DDS, other delivery systems, including fibroin NPs and SNEDDS, are also exploited and advanced for the treatment of IBD (Table 1). Epigallo-catechin 3-gallate (EGCG) is a natural polyphenol compound extracted from green tea [96, 97]. A large number of studies have shown that it has antioxidant and anti-inflammatory effects. [98]. Moreover, ECGC can relieve the symptoms of colonic inflammation and maintain the integrative structure of intestinal epithelial layer in IL-10 knockout mice [99, 100]. Despite this, its clinical translation has been constrained by limited bioavailability [97]. In a recent study, EGCG-loaded ovalbumin (OVA) NPs were facilely produced via self-assembling method and subsequently were used to treat UC mouse. In vivo results showed that the EGCG-loaded NPs EGCG increased the accumulation of EGCG in colitis tissue sites and showed significantly better therapeutic efficacy in alleviating UC than pristine EGCG [101].

**Table 1:** Summary of DDS of CM for the treatment of IBD

Type of DDS	Carrier	Experiment model	Encapsulated cargoes	Status	Therapeutic application and observations	References
pH-dependent DDS	Microsponges (Span 80, Eudragit L100 and PVA)	Acetic acid-induced rat colitis	Curcumin	In vivo	The curcumin loaded microsponges prevented the premature release of curcumin in upper GIT and specifically released the drug at colonic pH, thus a significant decrease in edema, necrosis, and hemorrhage of colon	[69]
	Hydrogel [poly(starch/acrylic acid)]	DSS-induced rat colitis	Rutin	In vivo	The rutin-loaded pH-responsive poly(starch/AAc) hydrogels offered maximum release as pH increased from pH 6.8 up to pH 7.7 in colon and attenuated mucosal injury in DSS-induced rat model of colitis	[70]
	NPs (Eudragit RL PO and Eudragit FS30D)	Acetic acid-induced rat colitis	Silybin	In vitro and in vivo	The Eudragit PL PO NPs of silybin decreased the most of the macroscopic and histological inflammation caused by acetic acid as much as dexamethasone	[60]
Microbiota-activated DDS	Ca-pectinate beads (pectin, CaCl_2_ and PEI)	Oxazolone-induced rat colitis	Resveratrol	In vivo	The Ca-pectinate bead formulation can be specifically degraded by the colonic bacterial enzyme and remarkably ameliorated oxazolone-induced colitis and cancer risk	[83]
	Coated pellets (chitosan, MCC, caffeic acid, hypromellose, alginic acid, zinc acetate and sodium alginate)	TNBS-induced rat colitis	Rutin	In vitro and in vivo	The rutin pellets coated with chitosan and sodium alginate reduced neutrophil infiltration, inflammatory response, and ameliorated clinical activity of TNBS-induced rats	[80]
	Microspheres (Ca-alginate, glutaraldehyde and chitosan)	TNBS-induced rat colitis	Icariin	In vitro and in vivo	Targeted microspheres loading icariin specifically released icariin in simulated colonic fluid, decreased colon mucosa damage index and reduced inflammatory responses	[85]
Other colon-targeted DDS	SNEDDS (Acrysol K-150, Capmul MCM and polyethylene glycol 400)	Chick embryo chorioallantoic membrane, acetic acid-induced IBD model in rats	Berberine	In vivo	The SNEDDS of berberine accumulated the drug at the site of inflammation, promoted the restoration of colonic mucosa and improved the crypt architecture	[106]
	Nanoemulsion (α-tocopherol, triacetin, or limonene, Tween 20 or Cremophor EL)	Healthy SD rats, indomethacin -induced mice small intestinal injury	Andrographolide (AG)	In vivo	AG-NE enhanced the relative bioavailability of AG, reduced the ulcer index and histological damage score of mice with indomethacin-induced intestinal lesions	[111]
	Ovalbumin NPs	LPS-activated Raw 264.7 macrophage, DSS-induced mice colitis	Epigallo-catechin 3-gallate (EGCG)	In vitro and in vivo	EGCG-NPs showed remarkably stronger capacity to suppress the secretion of pro-inflammatory mediators and promote the production of anti-inflammatory factor	[101]
Time-delay and pH-sensitive DDS	Colon-targeted tablet (ethyl cellulose and polyacrylic resin II and III)	Beagle dogs	Acetylharpagide	In vitro and in vivo	The acetylharpagide tablets caused delayed T_max_, prolonged absorption time, lower C_max_, and AUCINF obs	[75]
Co-delivery systems	pH-Sensitive NPs	TNBS-induced rat colitis	Curcumin and celecoxib	In vitro and in vivo	The pH-sensitive NPs of Cur-Cel restrained the release of encapsulated agents at pH of stomach and upper intestine and selectively released them at the colon region	[95]
	Composite microparticulates (CMPs) (thiolated chitosan/alginate and Eudragit S-100)	TNBS-induced rat colitis	Curcumin and 5-ASA	In vitro, ex vivo and in vivo	The pH-sensitive and mucoadhesive microparticles specifically delivered 5-ASA and curcumin to the colon and markedly alleviated the inflammation in colon of colitis rats	[94]
	SMEDDS	DSS-induced mice colitis	Curcumin and piperine	In vitro and in vivo	Cur-Pip-SMEDDS could target the injured epithelium of colon on DSS-induced colitis through retention enema administration, as shown by the reduction in DAI and histopathological lesion, and downregulating inflammatory mediators such as MPO activity, MDA content, as well as TNF α and IL-6 levels	[93]
	HA-coated PLGA/chitosan NPs	Colon-26 cell, raw 264.7 macrophage, Caco2-BBE, and DSS-induced mice colitis	Curcumin and siCD98	In vitro, ex vivo and in vivo	Orally administered HA-siCD98/CUR-NPs exhibited combinational effects against UC through protecting mucosal layer and alleviating inflammation	[92]

Resveratrol-loaded silk fibroin NPs (RL-FNPs) were prepared through precipitation and incubation methods to overcome its drawbacks. In this DDS, silk fibroin, as a good delivery carrier, harbors the anti-inflammatory and healing effects and could simultaneously enhance the efficacy of resveratrol. The anti-inflammatory effect of RL-FNPs was similar to that of dexamethasone [102]. Similar to resveratrol, quercetin, a potent anti-inflammatory, antioxidant agent [103] was encapsulated into the silk fibroin NPs and the antioxidant activity of quercetin was enhanced due to the synergistic effect of quercetin and silk fibroin [104].

**Table 2:** CM-based DDS for the treatment of IBD

Carrier	Experiment model	Encapsulated cargoes	Status	Therapeutic application and observations	References
NPs derived from ginger	DSS-induced colitis mouse model	–	In vitro and in vivo	Oral administration of GDNPs increased the survival and proliferation of IECs, reduced pro-inflammatory cytokines, and increased the anti-inflammatory cytokines in colitis models	[119]
NPs derived from ginger	Colon-26 and RAW 264.7 cells, normal mice	siCD98	In vitro and in vivo	Ginger-derived lipid vesicles can encapsulate siCD98 and a very low dose of siCD98 after oral administration specifically and efficiently reduced colonic CD98 gene expression	[120]
Angelica polysaccharide	TNBS-induced UC in rats	Dexamethasone (Dex)	In vivo	The Angelica polysaccharide-Dex conjugate greatly reduced the systemic immunosuppression caused by Dex and effectively conveyed Dex to the large intestine	[126]
Inulin	DSS-induced colitis mouse model	Budesonide	In vivo	The redox-sensitive NPs, based on amphiphilic inulin, specifically delivered budesonide to the inflamed colon tissue and exerted excellent therapeutic efficacy in comparison to drug suspension in colitis mice model	[131]
In situ self-spray coating system (DTPA dianhydride, SBC, SDS)	Caco-2 cells, raw 264.7 macrophage, DSS-induced rat colitis	Diallyl trisulfide (DATS)	In vitro and in vivo	Rectal administration of the DATS-loaded self-spray system produced exogenous H_2_S and suppressed the overproduction of pro-inflammatory cytokines, inhibited the adhesion of macrophages on the vascular endothelium, and repaired colonic inflamed tissues	[136]

Berberine (BR) is an isoquinoline alkaloid with a long history of medicinal use. BR is usually administered in a salt form for several clinical applications, such as anti-bacterial, anti-inflammatory, and gastrointestinal diseases [105]. However, poor stability and low bioavailability limited the application of BR for long time. Based on the solubility studies and the pseudo-ternary phase diagrams, SNEDDS of berberine was developed using Acrysol K-150, Capmul MCM and polyethylene glycol 400. Chick chorioallantoic membrane and in vivo efficacy assay individually revealed potent anti-angiogenic activity and anti-inflammatory effect of SNEDDS of BR [106]. Bruceine D (BD) is a natural quassinoid derived from B. javanica fruit and has various pharmacological activities including anti-cancer, anti-virus and anti-inflammatory effects [107]. However, the low solubility of BD is a barrier for its absorption and release, which results in poor bioavailability. To address this problem, SNEDDS of BD was developed and composed of MCT, solutol HS-15, propylene glycol. According to the pharmacokinetic statistics, pharmacokinetic parameters of BD-SNEDDS were enhanced as compared with BD-suspension. In addition, the colon length and body weight were significantly restored, and TLR4, NF-κB p65 protein expressions were suppressed in TNBS-induced UC rat model [108]. Different from SNEDDS, the composition of nanoemulsion (NE) increased water phase. Andrographolide (AG) is a natural diterpenoid isolated from *Andrographis paniculata*. Previous studies have shown that it has been utilized extensively for the treatment of IBD, because it can inhibit TNF-α, IL-1β, and NF-κB activities [109]. However, the low oral bioavailability seriously limited its application in the treatment of IBD [110]. AG-NE was prepared using high-pressure homogenization technique and composed of α-tocopherol, ethanol, cremophor EL, and water. In the pharmacokinetic assay, the bioavailability of AG from AG-NE was almost six times in comparison with that from the AG suspension [111].

Embelin is a benzoquinone derivative found in *Ardisia japonica*. It is reported that embelin targets microsomal prostaglandin E2 synthase (MPGES) and eicosanoid synthesizing proteins 5-lipoxygenase (5-LO), thus exhibiting potent anti-inflammation effect [112]. Embelin lipid nanospheres (LNs) were developed by hot homogenization followed by ultrasonication technique. Treatment with embelin LNs significantly reduced clinical activity and macroscopic scores compared to embelin conventional suspension in acetic acid-induced UC rat model. Meanwhile, MPO, lactate dehydrogenase (LDH) and lipid peroxides (LPO) levels were decreased and reduced glutathione (GSH) level was increased [113].

Anthocyanins are sugar conjugates of flavonoids and they are prevalent in flowers, fruits, and vegetables. Numerous studies have shown that anthocyanin-rich berries provide strong antioxidants and anticarcinogenic properties. However, poor bioavailability after oral consumption makes it difficult to reach sufficient concentration in the target sites. To increase the concentration of anthocyanins in the colon, three types of encapsulation systems, PA (pectin amide, CaCl_2_, glycerine), WPI (whey protein isolate) and SL (pectin amide, citric acid maltodextrin, schellac), were developed and evaluated in the simulated gastrointestinal fluid. Release result indicated that encapsulation can potentially stabilize anthocyanins in the GIT [114].

Three kinds of curcumin DDS, including SNEDDS, nanostructured lipid carrier (NLC) and lipid nanocapsules (NC) was conducted to treat IBD. Although the permeability of curcumin across Caco-2 cell monolayers in the NC group was better, another two groups could significantly reduce the secretion of TNF-α secretion in J774 cells. In vivo, decreased neutrophil infiltration and colonic inflammation were only found in the NLC group [115]. Another study prepared curcumin-loaded solid lipid microparticle (SLM) and investigated anti-inflammatory activity using rat colitis models. Rat treated with curcumin-loaded SLM showed faster weight gain and an increase of the whole colon length compared with DSS-induced rats [116].

## CM-based DDS for the treatment of IBD

### Ginger-derived DDS for the treatment of IBD

Ginger, derived from the rhizome of *Zingiber officinale* Rosc., is consumed as a spice and also used as an alternative medicine for a range of disorders like cold, fever, as well as many digestive tract problems like diarrhea and dyspepsia [117]. Studies have also shown that ginger and its active constituents, such as 6-gingerol and 6-shogaol, exhibit anti-oxidative, anti-inflammatory, and anti-cancer activities [118]. It is reported that a kind of NPs derived from edible ginger (GDNPs) exhibited efficient colon targeting following oral administration. GDNPs could also increase the survival and proliferation of intestinal epithelial cells (IECs), reduce the pro-inflammatory cytokines (TNF-a, IL-6 and IL-1β), and increased the anti-inflammatory cytokines (IL-10 and IL-22) in colitis models, suggesting that GDNPs has the potential to attenuate damaging factors while promoting the healing effect [119].

In addition, ginger-derived nanolipids are also developed as carrier material for the delivery of siRNA to treat UC. Ginger-derived lipid vesicles can encapsulate siCD98 and a very low dose of siCD98 after oral administration can specifically and efficiently reduce colonic CD98 gene expression [120]. Even the researcher suggested that siCD98/GDLVs have the potential to shift the current paradigm of siRNA delivery away from artificially synthesized NPs toward the use of nature-derived nanovectors from edible plants. 6-shogaol, the active ingredient of dried ginger, was loaded with folate-functionalized PLGA/poly-lactic acid (PLA) NPs using a versatile single step surface-functionalizing technique [121]. In vivo results showed that oral administration of NPs-PEG-FA/6-shogaol in a hydrogel system (chitosan/alginate) significantly alleviated colitis symptoms and accelerated colitis wound repair in DSS-treated colitis mice, by regulating the expression levels of pro-inflammatory (TNF-α, IL-6, IL-1β, and iNOS) and anti-inflammatory (Nrf-2 and HO-1) factors. Here, folic acid, a ligand for folate receptor which is overexpressed in IBD, was modified on the surface of the carrier to achieve colonic targeted delivery of the drug. In 2012, grape exosome-like NPs (GELNs) were proved by Zhang’s group that it can promote the intestinal tissue remodeling and protect against DSS-induced colitis [122]. Six years later, the group found that ginger exosome-like NPs (ELNs) are preferentially taken up by gut bacteria in an ELN lipid-dependent manner. ELN RNAs regulate gut microbiota composition and localization as well as host physiology, notably enhancing gut barrier function to alleviate colitis. More substances consisting of natural plants will be found and can be used for the treatment of IBD as therapeutic agents, drug carrier or both [123].

### CM-derived polysaccharide as drug carrier for the treatment of IBD

In recent years, polysaccharides are attracting more and more attention as a new class of colon targeting materials. Polysaccharides are usually not absorbed in the upper part of the digestive tract (stomach and small intestine), but can be specifically degraded by colon bacteria. As natural products, polysaccharides are not only cheap and easy to obtain, while their safety has been proven for long time and collected as a pharmaceutical excipient in various national pharmacopoeias [124]. Such compounds mainly include amylose, dextran, pectin, guar gum and chondroitin sulfate. In addition to the above polysaccharide macromolecules, other natural polysaccharides from CM were also developed to achieve colon-target drug delivery.

Angelica polysaccharide was extracted from fresh roots of *Angelica sinensis.* (Oliv.) Diels, which has been used as a CM to treat various diseases for thousands of years. There is mounting evidence revealing that Angelica polysaccharide possesses anti-ulcer and immunomodulation capabilities [125]. Zhou et al. chose Angelica polysaccharide as a drug carrier and succinate as a cross-linker, then dexamethasone (Dex)-polysaccharide conjugate was synthesized [126]. The newly synthesized dexamethasone-polysaccharide conjugate was found to greatly reduce the systemic immunosuppression caused by Dex and effectively convey Dex to the large intestine. Furthermore, the conjugate also showed promising therapeutic effect on TNBS-induced UC in rats.

*Psyllium*, viscous polysaccharides derived from the dried, ripe seeds of Plantago genus, has been reported as a medicinally active natural polysaccharide and used for the treatment of constipation, diarrhea, irritable bowel syndrome (IBS), UC, colon cancer, diabetes and hypercholesterolemia [127]. Due to its biodegradability and digestibility, *psyllium* is rapidly emerging as a low-cost drug carrier and has been verified in some researches [128]. Inulin, consisting of linear polydisperse chains of β(2,1) fructan, is another natural polysaccharide derived from natural plants. The presence of β(2,1) bonds in inulin prevents its degradation in the stomach and small intestinal. Abundance of hydroxyl groups makes it easy to interact with common coupling reagents [129]. A novel amphiphilic inulin by grafting it with a hydrophobic peptide can enable the resulting conjugate to self-assemble into nanostructures and then encapsulate therapeutic agents [130]. Another amphiphilic inulin derivative was developed by Sun et al. and was used to specifically deliver budesonide to the inflamed colon tissue [131]. Besides, *Ulva lactuca* (ULP) polysaccharide displays several biological features like lowering cholesterol, immunomodulatory and anti-heptotoxic property [132]. ULP can also stabilize the functional status of bio-membranes and act as an antioxidant compound and surfactant. Selenium (Se) is an essential micronutrient trace element and low Se status has been demonstrated in association with IBD progression. Se NPs (SeNPs) exert anti-inflammatory activity accompanied by low toxicity, especially when decorated with natural biological compounds. SeNPs decorated with *Ulva lactuca* polysaccharide (ULP) can mitigate body weight loss and colonic inflammatory damage on DSS-induced acute colitis in mice [133].

### CM as gas donors for the treatment of IBD

Gases such as nitric oxide, carbon monoxide and hydrogen sulfide play important roles in human physiological processes. Previous studies prepared styrene-maleic acid copolymer (SMA) micelles encapsulating tricarbonyldichlororuthenium (II) dimer (CORM2), a commonly used CO donor, and evaluated the therapeutic potential of SMA/CORM2 in DSS-induced inflammatory colitis murine model [134]. After treatment with SMA/CORM2 micelles, colitis symptoms (loss of body weight, diarrhea, and hematochezia) and histopathological colonic changes (shortening of the colon and necrosis or ulcers in the colonic mucosa) were significantly improved. It is reported that the first therapeutic-gas NO-responsive hydrogel was prepared and that it may prove useful in many applications, such as drug-delivery vehicles, inflammation modulators, and a tissue scaffold [135]. Hydrogen sulfide (H_2_S) is generated throughout the GI tract. Following colonic mucosal inflammation, the synthesis of H_2_S is significantly increased, accelerating the repair of ulcerative tissues, suggesting that H_2_S may function as an anti-inflammatory mediator. Diallyl trisulfide (DATS) is an oil-soluble sulfur compound of natural origin that is isolated from garlic. Theoretically, one molecule of DATS can produce three molecules of H_2_S in the presence of biological thiols, including GSH. However, owing to its insolubility in water, an ideal method of administering DATS has yet to be developed. As an exogenous H_2_S donor, a self-spray coating system that is derived from a DATS-loaded capsule with foaming capability is prepared. In vivo assay indicated that this system can suppress the overproduction of proinflammatory cytokines, inhibit the adhesion of macrophages on the vascular endothelium, and repair colonic inflamed tissues [136]. The representative examples of CM-based DDS for the treatment of IBD are showed in the Table 2.

## Conclusion and future perspectives

IBD, as a chronic inflammatory disease of the GIT, is common in young people and seriously affects the life quality of patients. As a long-standing cultural treasure, CM has always been favored by us. However, the unsatisfactory physical and chemical properties of CM have seriously affected their clinical application, and the emergence of various new DDS has solved these problems to some extent. The three major systems of active targeting, passive targeting, and tumor microenvironment-responsive DDS can also be mapped to the drug delivery strategy for IBD. The receptors that are known to be highly expressed in IBD are folate receptor, transferrin receptor, CD44 and CD98 glycoproteins, and more specific receptors are yet to be further explored. Secondly, the changes of various physiological conditions such as pH, temperature, oxygen and enzyme in the colon during the development of IBD are also worthy of further discussion. The existing theory is seriously lacking, which hinders the development of colon-targeted DDS.

One of the critical challenges in the IBD therapy is the drug-related adverse effects. The application of novel DDS into CM for the treatment of IBD has the potential to accumulate sufficient drug concentration at the disease region, enhance the solubility and bioavailability, prevent drug degradation, thus reducing the administration dose and systemic side effects and maximizing drug efficacy. Although various different functions DDS have been developed and investigated, further studies should be conducted to push good products into the clinic.

Ginger, as a member of CM, has been well developed for the treatment of IBD as therapeutic molecules, lipid nanovectors, and exosomal miRNAs, which could be a model CM worth learning. As an important signal molecule in the physiological process of human body, gas can be used as a therapeutic molecule, and can also be prepared as a gas-responsive DDS to deliver drugs. The components of plants that can be used as donors of such gas molecules are subject to our excavation and research. As a large class of molecules composed of CM, polysaccharides have long been unexplored as drug carriers. More plant polysaccharides as therapeutic drugs for IBD and CM delivery vehicles will greatly improve the safety and reduce the cost of treatment. For the treatment of IBD, the co-delivery of drugs and the design of prodrugs are also very promising development directions.

## Data Availability

Not applicable.

## Abbreviations

5-ASA: 5-aminosalicylic acid
CAC: colitis-associated cancer
CAM: complementary and alternative medicines
CD: Crohn’s disease
CM: Chinese medicine
DATS: diallyl trisulfide
DAI: disease activity index
Dex: dexamethasone
DDS: drug delivery system
DSS: dextran sulfate sodium
EGCG: epigallo-catechin 3-gallate
GIT: gastrointestinal tract
GSH: glutathione
HA: hyaluronic acid
IBD: inflammatory bowel disease
IBS: irritable bowel syndrome
IEC: intestinal epithelial cell
LDH: lactate dehydrogenase
LPO: lipid peroxides
MPO: myeloperoxidase
NPs: nanoparticles
P-gp: *P*-glycoprotein
PLA: poly-lactic acid
PLGA: poly (lactic-*co*-glycolic acid)
SASP: salicylazosulfapyridine
TNF: tumor necrosis factor
TNBS: 2,4,6-trinitrobenzenesulfonic acid
UC: ulcerative colitis
